# Supplementary material for: Spatial distribution and density of fibroblasts determine angiogenic response of endothelial cells
Source: Microfluid Nanofluidics. 2026 Oct 14;30(12):91. doi: 10.1007/s10404-026-02939-3 (PMC13668921; doi:10.1007/s10404-026-02939-3)
Supplement: Supplementary file 1 [file 10404_2026_2939_MOESM1_ESM.pdf]

Supporting Information

Spatial distribution and density of fibroblasts determine angiogenic response of endothelial cells

Pu Zhang[1,2], Melur K. Ramasubramanian[2*], Li-Heng Cai[1,3,4,5*]

Affiliations:

[1]Soft Biomatter Laboratory, Department of Material Science and Engineering, University of Virginia, Charlottesville, VA 22904, USA

[2]Department of Mechanical and Aerospace Engineering, University of Virginia, Charlottesville, VA 22904, USA

[3]Department of Chemical Engineering, University of Virginia, Charlottesville, VA 22904, USA

[4]Department of Biomedical Engineering, University of Virginia, Charlottesville, VA 22904, USA

[4]Department of Chemistry, University of Virginia, Charlottesville, VA 22904, USA

* Corresponding authors. M.R., mkr5a@virginia.edu; L.-H.C., liheng.cai@virginia.edu

The PDF file includes:

Figures S1-S4

Other Supplementary Materials for this manuscript include the following:

Movies S1

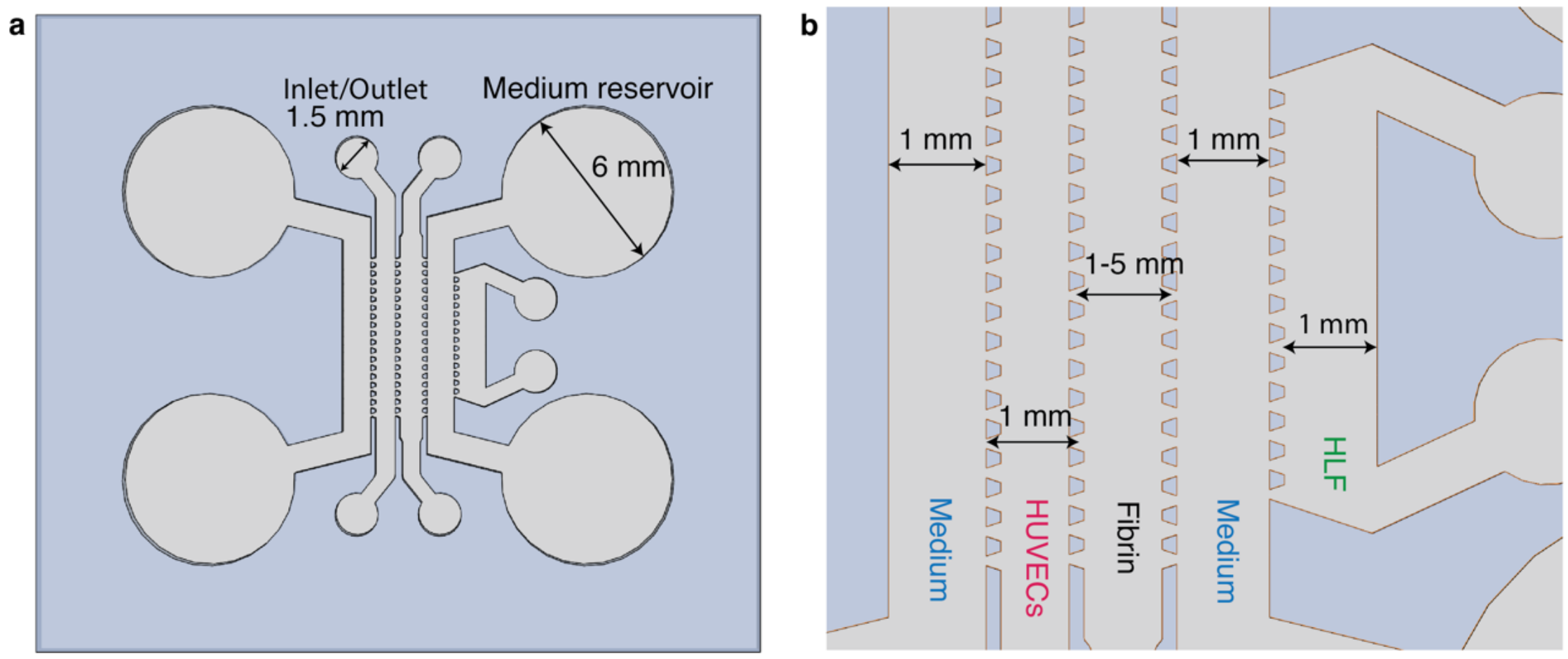

Figure S1. Dimensions and configurations of the microfluidic device. (a) The inlets and outlets of the device are 1.5 mm in diameter to facilitate efficient cell/gel seeding. The reservoir for cell culture medium is 6 mm in diameter. Each medium channel is designed with a height of 150 µm and a volume capacity of 34 µl, providing sufficient nutrients to support cell growth for 48 hours. **(b)** The channel configuration for vasculogenesis and angiogenesis includes 1,000 µm-wide cell and medium channels, separated by 130 µm-wide posts spaced 100 µm apart. We vary the width of the fibrin gel channel varies from 1 mm to 5 mm to study the impact of cell-to-cell distance on angiogenesis.

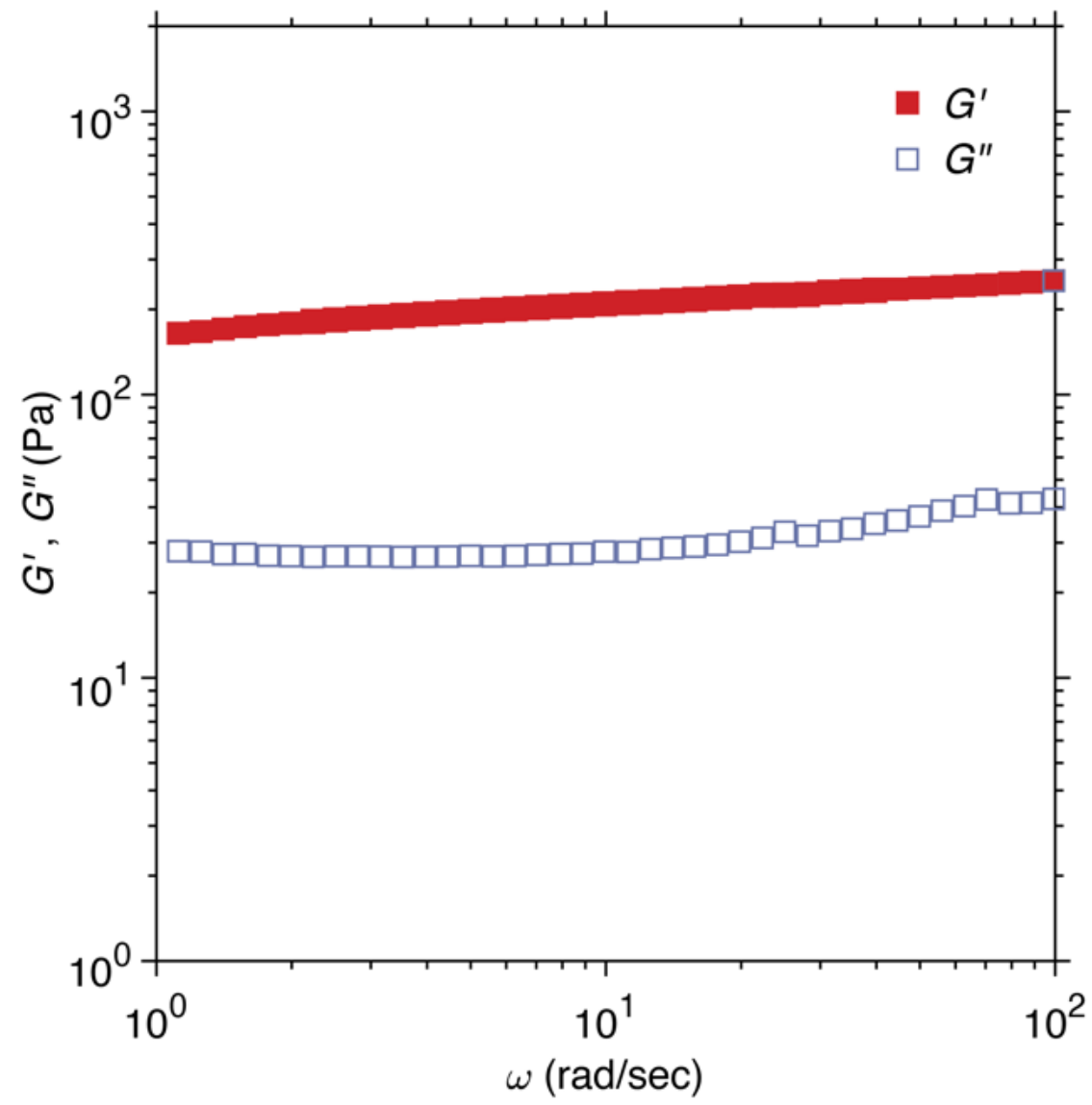

Figure S2. Dynamic mechanical properties of fibrin gel. Dependence of storage modulus (*G'*) and loss modulus (*G''*) of the fibrin gel (2.5 mg/mL) on oscillatory shear frequency ω. Over the entire frequency range, the fibrin gel has a nearly constant *G'* of 200 Pa and is nearly 10 times of the *G''*, suggesting the fibrin gel is an elastic solid. The measurement is performed at room temperature (20 °C) and 5% strain.

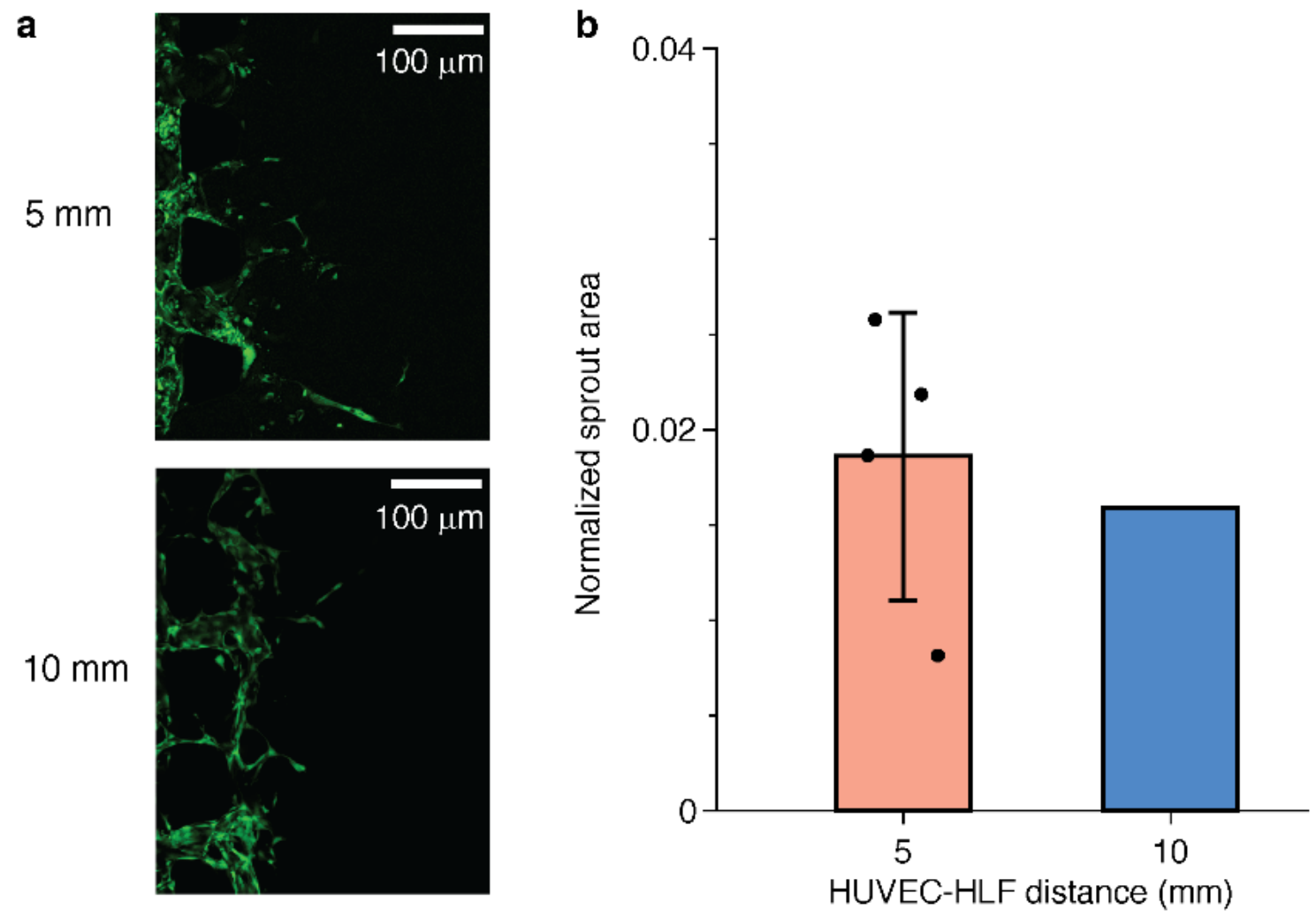

Figure S3. Angiogenic sprout area at a 10 mm HUVEC-HFL cell-cell distance. (a) Representative fluorescence confocal images of angiogenic sprouts at 5 mm and 10 mm cell-cell distances after being cultured for 120 hours. **(b)** Quantitative comparison of normalized sprout area between the 5 mm condition and a single measurement from the 10 mm condition.

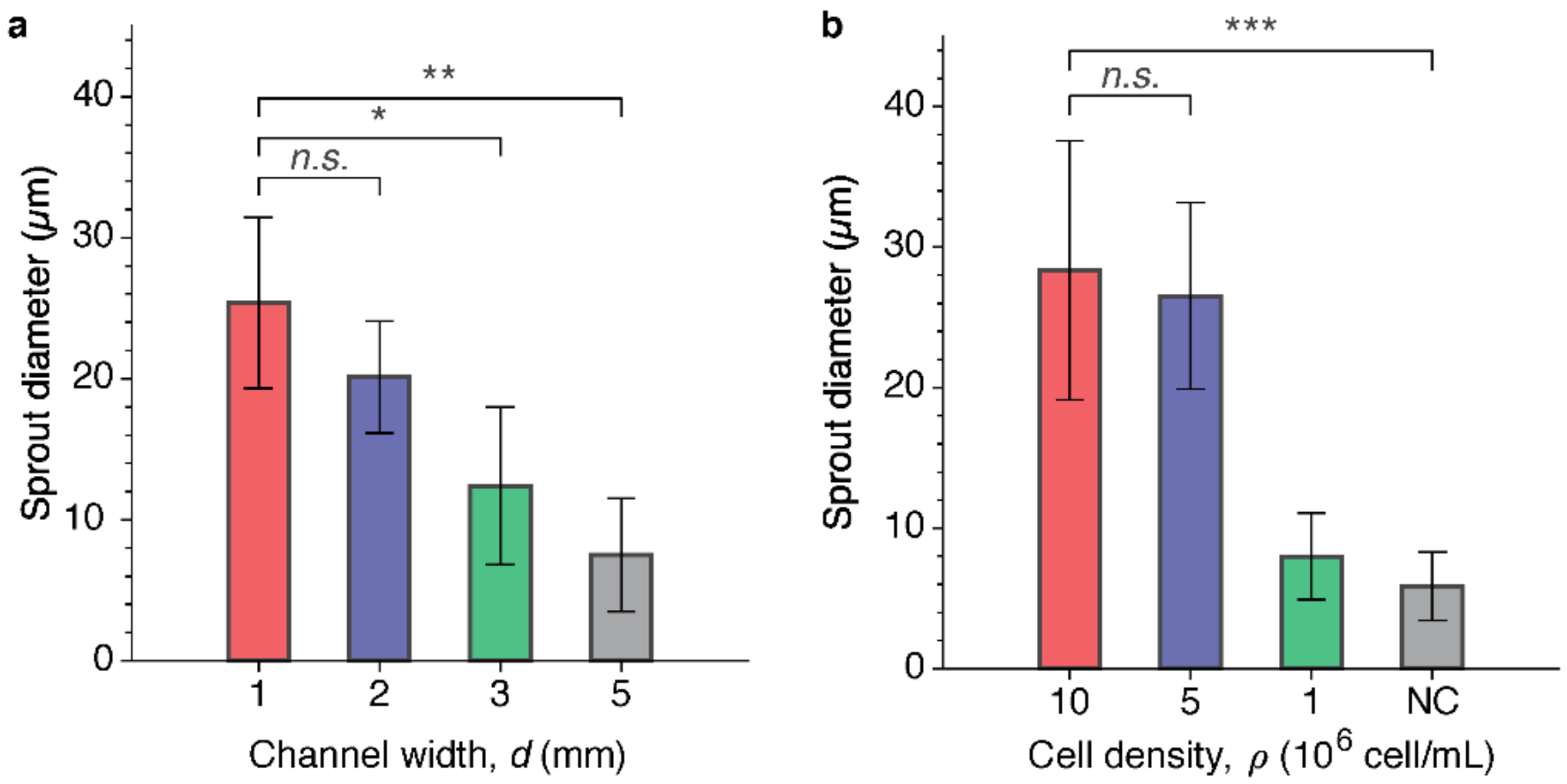

Figure S4. Sprout diameter at various cell-cell distances and fibroblast density. (**(a)** Sprout diameter at 120 hours with various cell-cell distances. **(b)** Sprout diameter at 120 hours with various fibroblast cell densities. The sprout diameter is measured at 120 hours using the diameter measurements plugin in ImageJ. 30 sprout diameters in channel 3G are acquired for each experimental sample and averaged to a single diameter value for that sample. For various cell-cell distances, the sprout diameter decreases significantly from an average of 20 μm to less than 8 μm after the distance exceeds 2 mm and reaches 5 mm. A similar trend is observed across various cell densities. Sprout diameter is consistent around 28 μm in samples with fibroblast cell seeding densities higher than 5×10^6 cells/ml. Reducing fibroblast density below 5×10^6 cells/ml, the sprout diameter is observed to be underdeveloped, measured at below 8 μm in average**.** *p<0.05; **p<0.01; ***p<0.001; *n.s.*, not significant; n=4.

Movie S1. The microbeads enter the vascular network through the medium channel and are visualized as they transport through the interconnected luminal structure, with their trajectories continuously tracked. The bead paths remain confined within the endothelial-lined lumina, with only a trace amount of microbeads leaking into the surrounding extracellular matrix.
